# An assessment of the eye care workforce in Enugu State, south-eastern Nigeria

**DOI:** 10.1186/1478-4491-7-38

**Published:** 2009-05-12

**Authors:** Boniface Ikenna Eze, Ferdinand Chinedu Maduka-Okafor

**Affiliations:** 1 Department of Ophthalmology, University of Nigeria Teaching Hospital, Ituku-Ozalla, Enugu, Nigeria

## Abstract

**Background:**

The availability and distribution of an appropriate eye care workforce are fundamental to reaching the goals of "VISION 2020: The right to sight", the global initiative for the elimination of avoidable blindness launched jointly by the World Health Organization and the International Agency for the Prevention of Blindness with an international membership of nongovernmental organizations, professional associations, eye care institutions and corporations. Periodic evaluation of these parameters is important in the journey towards achieving these goals. The objectives of the study were to determine the availability and distribution of human resources for eye care delivery in Enugu Urban, south-eastern Nigeria.

**Methods:**

The study was designed as a cross-sectional descriptive survey, the setting for which was all public and privately owned eye care facilities in Enugu Urban, Enugu State, south-eastern Nigeria, in October 2006. The health map of Enugu Urban and the hospital register of the Public Health Department of the Enugu State Ministry of Health were used to identify the eye health care facilities in Enugu Urban. A structured, pretested, researcher-administered questionnaire was used to capture data on cadre and distribution of the eye care personnel in these facilities.

Relevant population data were obtained from the Enugu Regional Office of the National Population Commission. Descriptive statistical analysis was used to generate percentages and proportions. Eye care personnel-to-population ratios were calculated and compared to World Health Organization recommendations.

**Results:**

Out of Enugu State's population of three million, Enugu Urban accounts for 22%. The population of Enugu Urban is distributed between the three-component Local Government Areas comprising Enugu North (31%), Enugu South (30%) and Enugu East (39%). There are 45 eye care facilities (public: 31 (69%); private: 14 (31%)) employing 252 eye care workers (public: 226 (90%); private: 26 (10%)) aged 18 to 63 (mean = 36.1 years, SD = 2 years) comprising males (36: 14%) and females (216: 86%), giving a male-to-female sex ratio of 1:6. The available eye care workforce is unevenly distributed between Enugu North (128: 51%), Enugu South (65: 26%) and Enugu East (59: 23%) Local Government Areas.

**Conclusion:**

Using broad and crude World Health Organization standards for minimum provider-to-population ratios, there is a sufficient eye care workforce in Enugu Urban. However, the maldistribution of the workforce creates a major barrier to uptake of eye care services. Policy modifications could reverse this maldistribution.

## Background

Primary Eye Care (PEC) is the provision of essential, affordable, accessible, practical and sustainable eye health care to the general population. PEC delivery uses the horizontal integration matrix model proposed by the World Health Organization (WHO) to incorporate PEC programmes into the existing Primary Health Care (PHC) structure [1,2]. Our definition of the eye care workforce includes all individuals who directly or indirectly provide care related to promotion, protection, and improvement of population eye health [3].

The eye care workforce has been identified as the bedrock of "VISION 2020: The right to sight", the global initiative for the elimination of avoidable blindness launched jointly by the World Health Organization and the International Agency for the Prevention of Blindness with an international membership of nongovernmental organizations, professional associations, eye care institutions and corporations. Vision 2020 offers the framework to define the appropriate, adequate, evenly distributed and satisfactorily motivated/remunerated eye care workforce to actualize the objectives of the programme [4]. In addition to the workforce, money, mobility, facilities (fixed and mobile) and management are the other complementary requirements for effective delivery of comprehensive eye care in the spirit of VISION 2020 [5].

Eye care includes promotive, preventive, curative or rehabilitative services; delivery locations include institution-based, community-based or both. There are three categories of eye care personnel: full-time eye care workers, integrated eye care workers and community-based eye care workers (medical and non-medical) [5,6]. The route of delivery and the type of eye care delivered are determined by public health needs, desired health impact, available resources and the prevailing socioeconomic environment [6].

In 1997, WHO established VISION 2020 recommendations for improvements in the eye care workforce in sub-Saharan Africa. By the year 2000, the region was expected to have the following eye care personnel-to-population ratios: one ophthalmologist per 500 000 people; one dispensing optician or optometrist per 500 000 people; one cataract surgeon/diplomate ophthalmologist per 250 000 people; one ophthalmic medical assistant or ophthalmic nurse per 500 000 people; one primary eye care trainer per one million people [7].

Unfortunately, however, economic reforms imposed by international monetary institutions on Nigeria, a debtor nation, resulted in budget restrictions that led to staff recruitment and development restrictions in all public sectors, including the health sector [8]. These measures negatively affected the health sector and its ability to offer eye care of quality. Eye care delivery, along with other ancillary services such as dental care, is subject to even tighter restrictions than more basic "life and death" services during periods of economic distress [3,5]. Restrictions in the public sector's ability to provide eye care have prompted some growth in private eye care services [9-11].

This study was conducted to measure the availability and distribution of human resources for eye care delivery in Enugu Urban, south-eastern Nigeria, especially in comparison to the VISION 2020 goals of WHO. Findings are intended to inform health policy formulators in monitoring and planning for further interventions in their existing programmes for prevention of blindness [9,12].

### Settings, data sources and methods

Enugu State is one of the 36 states of the Federal Republic of Nigeria; Enugu Urban is its administrative capital territory. Enugu State is divided into 17 Local Government Areas (LGAs); of these, three LGAs, comprising Enugu North, Enugu South and Enugu East, make up Enugu Urban. Enugu East has a comparatively significant rural component, as it was only recently carved out from the periphery of Enugu North LGA.

Geographically, Enugu State lies in the south-east of Nigeria, with a population of three million. The Enugu Urban population is about 707 000, distributed among Enugu North (31%), Enugu South (30%) and Enugu East (39%) [13].

Enugu State is located in the tropical rainforest climatic region, with patches of derived savannah. There are two seasons (rainy and dry) and the urban population is predominantly ethnic Ibos, although immigrants from other parts of the country also reside in the state [14]. The urban population is made up of mainly civil servants, traders, artisans and students/pupils of the various educational institutions in the state.

This is a descriptive cross-sectional survey of public and private eye health care facilities in Enugu Urban conducted between January and June 2006.

The health map of the three urban LGAs of Enugu North, Enugu South and Enugu East was obtained from their respective health departments, which provided information on the location of available eye health care facilities. Private facility data were obtained from the registry of private hospitals of the Public Health Department of Enugu State Ministry of Health. The State's Public Health Department also provided further information on cadre disposition of eye care personnel working in public or private eye care centres in Enugu State outside Enugu Urban.

Information on the types of in-service training programmes available for full-time eye care workers in public service was obtained from the Human Resources Department of Enugu State Ministry of Health. Relevant population figures based on projections from the 1991 census were obtained from the Enugu Zonal Office of the National Population Commission.

We visited each eye care facility to collect data on age, sex and cadre of eye care personnel in each facility by means of a structured, pretested, researcher-administered questionnaire.

Ancillary staff, such as drivers, laboratory workers and security men, and non-permanent staff – including interns and those doing their mandatory post-graduation, one-year National Youth Service – were excluded from the study.

### Data management

Data were analysed by means of the Statistical Package for Social Sciences (SPSS) software to generate percentage and proportions. Eye care personnel-to-population ratios were calculated and compared with WHO recommendations for VISION 2020.

### Ethics

Prior to commencement of this study, ethical clearance was sought and obtained from the Public Health Department of Enugu State Ministry of Health and the Enugu Zonal Office of the National Population Commission. Informed consent for participation was obtained from the research subjects by the researchers.

## Results

The population of Enugu State is three million. Of this, Enugu Urban has a population of 707, 000 (22%). The urban population is distributed among the LGAs as follows: Enugu North (218 000: 31%), Enugu South (210 000: 30%) and Enugu East (278 000: 39%).

There are 45 eye health care facilities in Enugu Urban, consisting of 31 (69%) public and 14 (31%) private facilities. Of the 31 public centres, two (6%) are tertiary, four (13%) are secondary and 25 (81%) are primary-level eye care centres.

Facility distribution by LGAs showed 20 (44%) in Enugu South: one cottage hospital, five health centres, two health clinics, eight private optometrist clinics, four private ophthalmologist clinics; in Enugu North 17 (38%): two university teaching hospital eye clinics, one cottage hospital, five health centres, three health clinics, five health posts, one private optometry clinic; in Enugu East, eight (18%): two cottage hospitals, five health centres, one private optometry clinic.

There were 252 eye care workers: 26 (10%) privately employed and 226 (90%) public employees. Of these, 36 (14%) were males and 216 (86%) were females, giving a male-to-female ratio of 1:6. The age range was 18 to 63 (mean = 36.1 years, SD = 2 years). The age and sex distribution of workers are shown in Table 1.

**Table 1:** Age and sex distribution of eye care workers

**Age (years)**	**Sex**		**Total**
		
	M	F	
10–20	1	15	16

21–30	1	55	56

31–40	11	74	85

41–50	10	34	44

51–60	11	34	45

61–70	2	4	6

**Total**	**36**	**216**	**252**

Source: Eye care workforce survey in Enugu State, 2006

The distribution of staff by cadre and LGA is shown in Table 2. The comparison of eye care personnel-to-population ratios with WHO recommendations is shown in Table 3.

**Table 2:** Cadre and distribution of eye care workers by LGA

**Cadre of personnel**	**Enugu North** **n (%)**	**Enugu South** **n (%)**	**Enugu East** **n (%)**	**Total (Enugu Urban)** **n (%)**
Ophthalmologist				
• Fellow	13 (77)	4 (24)	0 (0.0)	17 (100)
	
• Diplomate	0 (0.0)	0 (0.0)	0 (0.0)	0 (100)

Optometrist	9 (50)	8 (44.4)	0 (0.0)	17 (100)

Ophthalmic nurse	23 (100)	0 (0.0)	0 (0.0)	23 (100)

Medical officer	6 (50)	4 (33.3)	2(16.7)	12 (100)

Staff nurse/midwife	26 (50)	13 (25)	13 (25)	52 (100)

Community health officer	7 (37)	6 (32)	6 (32)	19 (100)

Community health extension worker	36 (34)	30 (28)	40 (38)	106 (100)

Primary eye care trainer	5 (100)	0 (0.0)	0 (0.0)	5 (100)

**Total (%)**	**125 (51)**	**65 (26)**	**61 (25)**	**251 (100)**

Source: Eye care workforce survey in Enugu State, 2006

**Table 3:** Eye care personnel-to-population ratios by LGA compared with WHO recommended ratios

	**Enugu North**	**Enugu South**	**Enugu East**	**Total (Enugu Urban)**	**WHO recommended ratio**
Population	218 000	210 000	278 000	707 000	--

Ophthalmologist	1:17 000	1:53 000	0:278 000	1:42 000	1:500 000

Optometrist	1:24 000	1:26 000	1:278 000	1:39 000	1:500 000

Ophthalmic nurse	1:9 000	0.210 000	0.278 000	1:28 000	1:400 000

Primary eye care trainer	1:36 000	0:210 000	0.278 000	1:118 000	1: 1 000 000

Community health extension worker	1:6 000	1:7 000	1:8 000	1:7 000	1:1 000 000

Source: Eye care workforce survey in Enugu State, 2006

There is not a single ophthalmologist (fellow or certified specialist – diplomate), cataract surgeon or ophthalmic nurse working in either the public or private sector in Enugu State outside Enugu Urban, although there are four optometrists (all in private practice) outside Enugu Urban.

The available in-service training programmes for public eye care workers include workshops, update courses, refresher courses and bonded scholarship for employees seeking full-time, long term, in-service training usually lasting for one year or longer.

The bonded scholarship programme is usually chosen by staff nurses/midwives for their one-year ophthalmic nursing training and by medical officers going for residency training in ophthalmology.

There is no available training programme for cataract surgeons and diplomate ophthalmologists.

In Enugu State there are eight health training institutions open to qualified applicants from the public: two university teaching hospitals, one national orthopaedic hospital, one federal neuropsychiatric hospital, two schools of health technology and two schools of nursing/midwifery.

Referrals, usually through written referral letters, originate from lower to higher-level eye care centres.

## Discussion

Taken as a whole, the eye care workforce in Enugu Urban and Enugu State would appear to be adequate by WHO standards [7]. However, the gross maldistribution of the available eye care personnel among the three component LGAs in Enugu Urban and between Enugu Urban and its rural population is a cause for serious public eye health concern. This maldistribution also affects the private health sector. This runs contrary to the fundamental principle of fair and even distribution of available human resources for eye care delivery as established by VISION 2020 [3].

Similar maldistribution patterns of the available eye care workforce have been reported elsewhere from other developing countries similar to Nigeria [3,8,15-19]. Nwosu [20], Quarcopoome [21], Katung [22], Abiose [23] and Eze et al. [24] have also reported similar trends in the distribution of the available eye care workforce and highlighted the resultant problems of economic and geographical barriers to uptake of eye care if not urgently addressed. It has been established that opportunities for professional development/advancement and living conditions/availability of social amenities are more important determinants of health worker mobility than remuneration [3,25]. In the present report, enhanced career progression opportunities, availability of social amenities and higher prospects for lucrative part-time private practice in Enugu Urban are implicated in fuelling this maldistribution.

In Enugu, we observed WHO's recommended pyramid-style distribution of eye care cadres, in which lower-cadre workers are more numerous at the base, while higher-cadre workers are fewer and located at the apex [1,7]. This observation agrees with various ophthalmic workforce survey results previously reported [13,19,23]. However, in Enugu, we noted an absolute dearth of middle-level ophthalmic workforce (cataract surgeons and diplomates), likely due to the local lack of training programmes for these cadres of eye care workers.

Consistent with the trend worldwide, the sex distribution of the available eye care workforce shows a preponderance of females over males (85.7% versus 14.3%), with a male-to-female ratio of 1:6. This is in keeping with WHO report that the majority (70% to 80%) of the world's health care workers are females [18]. This has negative implications for workforce mobility, since married females in Africa are less mobile than their male counterparts, secondary to their gender-related domestic functions.

The age distribution shows that a majority (62%) of the eye care health workers in our study are 40 years of age or younger. This implies a long-term temporal stability of the available eye care workforce, since a large proportion of eye care personnel still have many productive working years before the mandatory retirement age of 60 years in Nigeria.

## Conclusion

In the aggregate, Enugu Urban seems to have an adequate eye care workforce to deliver essential eye care. Unfortunately, there exists a worrisome maldistribution of the available eye care workforce in Enugu Urban and the rural population of Enugu State. This is a fundamental departure from the principle of equal/universal access to eye care as enshrined in the VISION charter.

The apparent sufficiency of eye care workforce in Enugu Urban suggested by the findings of this survey underscores a major shortcoming of using VISION 2020/WHO recommendations for eye care personnel-to-population ratios in assessing eye care workforce availability. The authors suggest a review of the VISION 2020/WHO recommendations to give appropriate weighting to such variables as population size/density and spatial distribution of available eye care personnel. The results of our study suggest that health policy-makers should schedule periodic staff audits of eye care workers in Nigeria's districts to create the data set that would allow redistribution of the available human resources for eye care delivery.

In the absence of any cataract surgeon or diplomate ophthalmologist in the region, policy-makers may want to consider training the middle-level ophthalmic workforce to provide those services. Specifically, interested potential trainees should be sponsored for training while arrangements for local establishment of the training programme are made. Additionally, to retain the current crop of rural eye care workers and possibly attract more, policy formulators should address the rural eye care workers' limited access to professional development and career progression by creating scholarship programmes and giving preferential sponsorship to workshops/refresher courses specifically for rural eye care workers.

Furthermore, the authors advocate intersectoral cooperation between health and other relevant sectors to provide basic social amenities where health facilities are located. Clearly, in the limited-resource setting of Enugu State, encouraging private eye care providers to fill the vacuum created by this maldistribution is not a viable public health option; private eye care services are exorbitant and often inaccessible to the poor. Finally, fair and even distribution of the available eye care workforce should have an overriding influence over other factors when making job postings in the public health sector.

## Competing interests

The authors declare that they have no competing interests.

## Authors' contributions

BIE conceptualized the research, designed the study protocol, participated in data collection, analysis and interpretation and wrote the initial draft of the manuscript. FCMO participated in data collection, analysis and interpretation; made substantial intellectual input into the manuscript; and approved the final version.
